# Supplementary material for: Association between the Immunophenotype of Peripheral Blood from mCRPC Patients and the Outcomes of Radium-223 Treatment
Source: Diagnostics (Basel). 2023 Jun 29;13(13):2222. doi: 10.3390/diagnostics13132222 (PMC10340498; doi:10.3390/diagnostics13132222)
Supplement: Supplementary file 1 [file diagnostics-13-02222-s001.zip › diagnostics-2480425-supplementary.pdf]

Supplementary Table S1. Demograhic, clinical and blood characteristics of mCRPC patients stratified in slow and fast progression.

	**Slow Progression (*n* = 7)**	**Fast Progression (*n* = 5)**
Age	76.43 ± 7.39	82.80 ± 6.94
Weight (kg)	77.00 ± 10.39	71.60 ± 9.88
Height (cm)	171.00 ± 5.72	164.8 ± 9.01
Months between diagnosis and sample collection	83.14 ± 39.08	115.00 ± 100.10
Months between resistance to castration and sample collection	62.43 ± 43.59	33.60 ± 21.66
Month between metastasis and sample collection	72.29 ± 43.15	42.80 ± 30.82
Stage disease at moment of diagnosis:	I (14%); II (0%); III (14%); IV (72%)	I (20%); II (40%); III (0%); IV (40%)
Gleason score:		
≤8	60%	71%
≥9	40%	29%
ECOG performance-status score (0–2):		
1	4	3
2	3	2
Median haematological and biochemical values:		
Platelets (×109/L)	248.60 ± 102.10	194.80 ± 67.27
Lymphocytes (×109/L)	1.462 ± 0.368	1.03 ± 0.36
Neutrophils (×109/L)	5.82 ± 3.65	5.92 ± 2.63
Monocytes (×109/L)	0.43 ± 0.13	0.64 ± 0.16
Hemoglobin (g/L)	100.70 ± 16.95	109.00 ± 9.72
Albumin (g/L)	38.30 ± 3.57	40.82 ± 2.02
Alkaline phosphatase (U/L)	248.00 ± 188.20	164.00 ± 166.40
PSA (μg/L)	333.20 ± 411.90	96.98 ± 118.10
Lactate dehydrogenase (U/L)	249.60 ± 56.66	277.00 ± 86.66
Previous treatment:		
Abiraterone (yes/no)	7/0	4/1
Enzalutamide (yes/no)	6/1	4/1
Chemotherapy (yes/no)	6/1	3/2

Values are presented as mean±SD. Gleason score and ECOG were referred at moment of sample collection. PSA: Prostate-specific antigen.

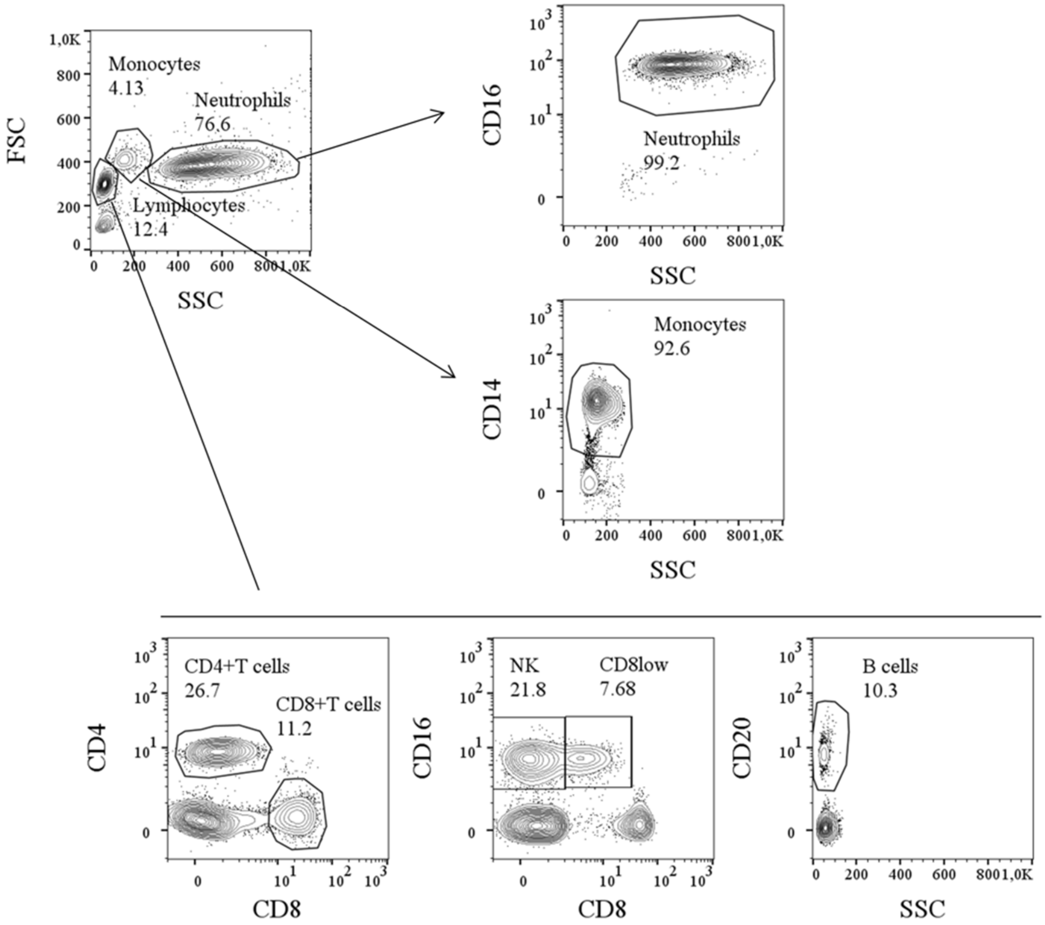

Supplementary Figure S1. Gating strategy by flow cytometry to detect different blood populations as described in Material and Methods.

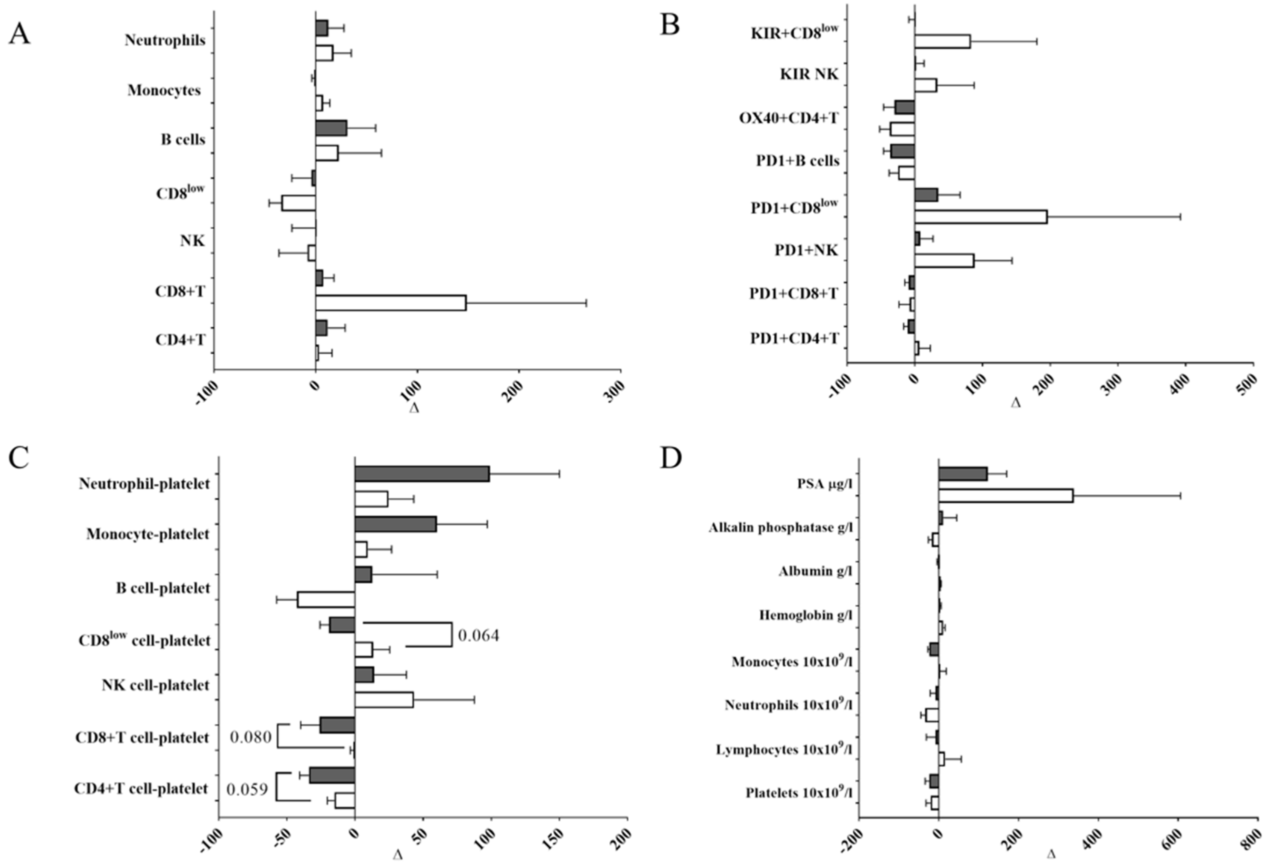

Supplementary Figure S2. Increment or decrement percentage between two weeks after the second Radium-233 injection and baseline: (**A**) leukocyte populations; (**B**) immunomodulatory molecules; (**C**) platelets—leukocytes complexes; (**D**) biochemical analytes based on slow (white) and fast (grey) progression. The Mann-Whitney test was used for the comparison of independent variables. *P*-values <0.05 were considered significant.
